# Calcium Sulphide as a Prophylactic

**Published:** 1906-11

**Authors:** A. T. Cuzner

**Affiliations:** Gilmore, Fla.


### CALCIUM SULPHIDE AS A PROPHYLACTIC.

By A. T. CUZNER, M.D.,

GILMORE, FLA.

I was very much pleased with a recent article on calcium sulphide as a prophylactic against malarial and yellow fever infection. From my personal observations during the yellow fever epidemic of 1888, in Jacksonville, Fla., supplemented by experimental experience since, I am satisfied we have discovered an extremely valuable preventive and aid in the cure of these diseases. The author says in part: "It is claimed that when the human body has become so saturated with this drug that the sulphide odor may be detected on the breath and in the perspiration, no known micro-organism can exist in the body; furthermore, that no insect, mosquito, flea, fly, pediculus, jigger, redbug, bedbug, or any other insect pest, will bite or remain upon the person of an individual as long as he is thus saturated." Conceding the position that both malaria, in all its forms, and yellow fever are both caused by micro-organisms of a very low order of vitality, but who have the power, notwithstanding, of endangering the health and life of the patients they infect, it is reasonable to suppose that such agents as sulphur and calcium (great oxidizers separated or combined) should have a destructive effect on these lowest known organisms. Let us look into the subject more closely.

Hueppe, The Principles of Bacteriology, tells us, page 50, "That microbes or micro-organisms can live without free oxygen—that is, without the oxygen of the air. Oxygen is taken from the vital chemical compounds of the body (of a low order of vitality) which, in consequence suffer disintegration." Again he says: "Cholera cultures are virulent when young, first becoming impotent through access of air."

In this last statement there is much food for thought, but we refrain, as it is a little aside from our subject.

On page 259, Hueppe says further: "Disease-producing bacteria may affect man in very different ways. They may cause changes by growing and multiplying in vital organs and through thus altering the metabolism of important tissues may influence unfavorably the metabolism of the whole body; or they may rob the body of important nutrient material and introduce products of their own metabolism into the body of their host; or they may in the act of satisfying their own need of energy rob from the body certain elements or substances needed by it; or they may generate poisons in their own bodies and, like poisonous plants, be in themselves poisons." Reasoning from the above quotations as a basis, and from the facts resulting from the use of calcium sulphide as a preventive, I assume as a temporary hypothesis, that this remedy is chiefly meritorious from its known oxidating power over bacteria and thereby depriving them of the power of proliferation and the working of evil to the normal tissues of the body. We have found highly pulverized sulphur of commerce dusted on the human tonsils has great curative power over tonsillitis. The subject of disinfecting and rendering the body aseptic to many diseases by the use of sulphide of calcium in excess, is well worthy the attention of the general practitioner. That we have an abundance of remedies for the destruction of most, if not all the known bacteria, goes without saying, but then these remedies are in themselves harmful to the human body, hence not available for extended use.

---

THE epidemic of anchylostomum duodenale, by which the western part of Germany, the mining district of Westphalia, suffered very much a few weeks ago, has led to interesting results. The anemic condition is soon followed by an affection of the nervous system, epileptic attacks, paralysis and cardiac diseases. The warmer the mines were, the more the miners were affected, the best temperature being about 22° C. Sanitary control of the patient and destruction of the feces have arrested the disease.—*Therapeutic Gazette.*

---

MARKED tenderness over the lower end of the radius, after traumatism, without deformity, is suspicious of fissure of the bone. Mobility or crepitus may be obtainable.—*American Journal of Surgery.*

---

IN determining whether or not to operate after injuries to the head, a surgical judgment of the case is usually better than one based strictly on the application of neurological rules.—*American Journal of Surgery.*

---

"EGGSHELL crackle" elicited in palpating a tumor of the cranial bones is diagnostic of sarcoma originating in the diploë.—*American Journal of Surgery.*

